# Photosynthetic Induction Under Fluctuating Light Is Affected by Leaf Nitrogen Content in Tomato

**DOI:** 10.3389/fpls.2022.835571

**Published:** 2022-02-17

**Authors:** Hu Sun, Yu-Qi Zhang, Shi-Bao Zhang, Wei Huang

**Affiliations:** ^1^Kunming Institute of Botany, Chinese Academy of Sciences, Kunming, China; ^2^University of Chinese Academy of Sciences, Beijing, China; ^3^Institute of Environment and Sustainable Development in Agriculture, Chinese Academy of Agriculture Sciences, Beijing, China

**Keywords:** fluctuating light, nitrogen, photosynthesis, mesophyll conductance, photosynthetic limitation

## Abstract

The response of photosynthetic CO_2_ assimilation to changes of illumination affects plant growth and crop productivity under natural fluctuating light conditions. However, the effects of nitrogen (N) supply on photosynthetic physiology after transition from low to high light are seldom studied. To elucidate this, we measured gas exchange and chlorophyll fluorescence under fluctuating light in tomato (*Solanum lycopersicum*) seedlings grown with different N conditions. After transition from low to high light, the induction speeds of net CO_2_ assimilation (*A*_*N*_), stomatal conductance (*g*_*s*_), and mesophyll conductance (*g*_*m*_) delayed with the decline in leaf N content. The time to reach 90% of maximum *A*_*N*_, *g*_*s*_ and *g*_*m*_ was negatively correlated with leaf N content. This delayed photosynthetic induction in plants grown under low N concentration was mainly caused by the slow induction response of *g*_*m*_ rather than that of *g*_*s*_. Furthermore, the photosynthetic induction upon transfer from low to high light was hardly limited by photosynthetic electron flow. These results indicate that decreased leaf N content declines carbon gain under fluctuating light in tomato. Increasing the induction kinetics of *g*_*m*_ has the potential to enhance the carbon gain of field crops grown in infertile soil.

## Introduction

Plants capture light energy to produce chemical energy ATP and NADPH, which are used to drive nitrogen assimilation and the conversion of CO_2_ to sugar. Enhancing net CO_2_ assimilation rate (*A*_*N*_) is thought to be one of the most important targets for improving plant growth and crop productivity (Kromdijk et al., 2016; Yamori et al., 2016a; South et al., 2019; Ferroni et al., 2020). Many previous studies indicated that increasing *A*_*N*_ under constant high light can boost plant biomass (Kebeish et al., 2007; Timm et al., 2012, 2015). Recently, some studies reported that the response of *A*_*N*_ to the increases of illumination significantly affects the carbon gain and thus influences plant growth (Slattery et al., 2018; Adachi et al., 2019; Kimura et al., 2020; Yamori et al., 2020; Zhang et al., 2020). Therefore, altering the photosynthetic performance under dynamic illumination is a promising way to improve photosynthesis under natural fluctuating light (FL) conditions.

Plants grown under high nitrogen (N) concentration usually have higher biomass than plants grown under low N concertation (Makino, 2011). An important explanation for this is that leaf photosynthetic capacity is related to the leaf N content in many higher plants (Yamori et al., 2011; Fan et al., 2020; Li et al., 2020), since stromal enzymes and thylakoid proteins account for the majority of leaf N (Makino and Osmond, 1991; Sudo et al., 2003; Takashima et al., 2004). Furthermore, stomatal conductance (*g*_*s*_) and mesophyll conductance (*g*_*m*_) under constant high light are also increased in plants grown under high N concentration, which speeds up CO_2_ diffusion from atmosphere to chloroplast carboxylation sites and thus favors the operation of *A*_*N*_ under constant high light (Yamori et al., 2011). However, few is known about the effects of leaf N content on non-steady-state photosynthetic performances under FL.

Under natural field conditions, light intensity exposed on leaf surface dynamically changes on timescales from milliseconds to hours (Pearcy, 1990; Slattery et al., 2018). Furthermore, FL and N deficiency usually occurs concomitantly, but how FL and N deficiency interacts to influence photosynthetic physiology in crop plants is poorly understood. After a sudden transitioning from low to high light, the gradual increase of *A*_*N*_ is termed “photosynthetic induction.” Recent studies indicated that the induction response of *A*_*N*_ was significantly affected by the induction speed of *g*_*s*_ (De Souza et al., 2020; Kimura et al., 2020). Gene expression plays a crucial role in the induction response of *g*_*s*_ under FL. For example, the *slow anion channel-associated 1* (*slac1*), *open stomata 1* (*ost1*) and abscisic acid-deficient *flacca* mutants, and the *proton ATPase translocation control 1* (*PATROL1*) overexpression line had faster stomatal opening responses than WT types in *Arabidopsis thaliana*, rice and tomato (De Souza et al., 2020; Kaiser et al., 2020; Kimura et al., 2020; Yamori et al., 2020). Furthermore, the stomatal opening during photosynthetic induction can be affected by environment conditions such as drought, target light intensity, magnitude of change, *g*_*s*_ at low light, the time of day, and vapor pressure deficit (Zivcak et al., 2013; Kaiser et al., 2020; Sakoda et al., 2020; Eyland et al., 2021). However, there have been few studies that examined the effect of leaf N content on the induction response of *g*_*s*_ after transition from low to high light (Li et al., 2020).

In addition to *g*_*s*_, *g*_*m*_ is a major factor that affects CO_2_ concentration in chloroplast, because *g*_*m*_ determines the CO_2_ diffusion from intercellular space into the chloroplast (Flexas et al., 2013; Carriquí et al., 2015). In general, *g*_*m*_ can be determined by structure across leaf profiles, genetic types, biochemical components, and environmental conditions (Yamori et al., 2011; Xiong et al., 2015; Théroux-Rancourt and Gilbert, 2017; Ferroni et al., 2021). Previous studies have highlighted that *g*_*m*_ is the most important limiting factor for *A*_*N*_ in many angiosperms (Peguero-Pina et al., 2017; Xiong et al., 2018; Yang Z.-H. et al., 2018, Yang et al., 2021; Gago et al., 2020). Short-term response of *g*_*m*_ to light intensity has been determined and found that it varies between plant species (Tazoe et al., 2009; Yamori et al., 2010a; Xiong et al., 2018; Yang et al., 2020). However, the induction response of *g*_*m*_ after transition from low to high light is less known. The *g*_*m*_ level under constant light is also significantly affected by leaf N content (Yamori et al., 2011). Furthermore, the rapid responses of *g*_*m*_ to CO_2_ concentration and temperature were also affected by leaf N content (Xiong et al., 2015). However, no studies have elucidated the effect of leaf N content on induction response of *g*_*m*_ upon transfer from low to high light.

In this study, we aimed to characterize the effects of leaf N content on induction kinetics of *A*_*N*_, *g*_*s*_, and *g*_*m*_ after a sudden transition from low to high light. Gas exchange and chlorophyll fluorescence were measured in tomato plants grown under contrasting N concentrations. The dynamic limitations of *g*_*s*_, *g*_*m*_, and biochemical factors imposed on *A*_*N*_ were analyzed based on the biochemical model for C3 photosynthesis (Farquhar et al., 1980). The effects of leaf N content on photosynthetic performances during photosynthetic induction were revealed.

## Materials and Methods

### Plant Materials and Growth Conditions

Tomato (*Solanum lycopersicum* cv. Hupishizi) plants were grown in a greenhouse with the light condition of 40% full sunlight. The day or night air temperatures were approximately 30 or 20°C, the relative air humidity was approximately 60–70%, and the maximum light intensity exposed to leaves was approximately 800 μmol photons m^–2^ s^–1^. Plants were grown in 19-cm plastic pots with humus soil, and the initial soil N content was 2.1 mg/g. Plants were fertilized with Peters professional water solution (N:P:K = 15:4.8:24.1, quality ratio) or water as follows: high nitrogen (HN, 0.15 g N/plant every 2 days), middle nitrogen (MN, 0.05 g N/plant once a week), and low nitrogen (LN, 0 mM N/plant). The fertilizer was dissolved in 0.3% water solution and subsequently was used for fertilization, and the nitrogen sources were 24% (NH_4_)_3_PO_4_, 65% KNO_3_, and 9.5% CH_4_N_2_O. To prevent any water stress, these plants were watered every day. After cultivation for 1 month, youngest fully developed leaves were used for measurements. For each N treatment, five leaves form five independent plants were used for gas exchange and chlorophyll fluorescence measurements.

### Gas Exchange and Chlorophyll Fluorescence Measurements

An open gas exchange system (LI-6400XT; Li-Cor Biosciences, Lincoln, NE, United States) was used to simultaneously measure gas exchange and chlorophyll fluorescence. Measurements were taken at a leaf temperature of approximately 25°C, leaf-to-air vapor pressure deficit of 1.2–1.4 kpa, and flow rate of air through the system of 300 mmol min^–1^. To measure photosynthetic induction after a short-term shadefleck, leaves were first adapted to a light intensity of 1,500 μmol photons m^–2^ s^–1^ and air CO_2_ concentration of 400 μmol mol^–1^ for > 20 min until *A*_*N*_ and g_*s*_ reached steady state. Then, leaves were subjected to 5 min of low light (50 μmol photons m^–2^ s^–1^) followed by 30 min of high light (1,500 μmol photons m^–2^ s^–1^), and gas exchange and chlorophyll fluorescence were logged every minute. iWUE was calculated as iWUE = *A*_*N*_/*g*_*s*_. The relative *A*_*N*_, *g*_*s*_, and *g*_*m*_ curves were obtained from the standardization against the maximum values after 30 min photosynthetic induction at high light. The time required to reach 90% of the maximum *A*_*N*_, *g*_*s*_, and *g*_*m*_ was estimated by the first time at which the relative values were higher than 90%. After photosynthetic induction measurement, the response of CO_2_ assimilation rate to incident intercellular CO_2_ concentration (*A*/*C*_*i*_) curves was measured by decreasing the CO_2_ concentration to a lower limit of 50 μmol mol^–1^ and then increasing stepwise to an upper limit of 1,500 μmol mol^–1^. For each CO_2_ concentration, photosynthetic measurement was completed in 3 min. Using the *A*/*C*_*i*_ curves, the maximum rates of RuBP regeneration (*J*_*max*_) and carboxylation (*V*_*cmax*_) were calculated (Long and Bernacchi, 2003).

The quantum yield of PSII photochemistry was calculated as Φ_*PSII*_ = (*F_*m*_*′-*F*_*s*_)/*F_*m*_*′ (Genty et al., 1989), where *F_*m*_*′ and *F*_*s*_ represent the maximum and steady-state fluorescence after light adaptation, respectively (Baker, 2004). The total electron transport rate (ETR) through PSII (*J*_*PSII*_) was calculated as follows (Krall and Edwards, 1992):


(1) $$J_{\text{PSII}}=\phi_{\text{PSII}}\times \text{PPFD}\times L_{\text{abs}}\times 0.5$$

where PPFD is the photosynthetic photon flux density, and leaf absorbance (*L*_*abs*_) is assumed to be 0.84. We applied the constant of 0.5 based on the assumption that photons were equally distributed between PSI and PSII.

### Estimation of Mesophyll Conductance and Chloroplast CO_2_ Concentration

Mesophyll conductance was calculated according to the following equation (Harley et al., 1992):


(2) $${\text{g}}_{\text{m}}=\frac{A_{\text{N}}}{C_{\text{i}}-\Gamma^{*}\,\left(J_{\text{PSII}}+8\,\left(A_{\text{N}}+R_{\text{d}}\right)\right)/\left(J_{\text{PSII}}-4\,\left(A_{\text{N}}+R_{\text{d}}\right)\right)}$$

where *A*_*N*_ represents the net rate of CO_2_ assimilation; *C*_*i*_ is the intercellular CO_2_ concentration; Γ* is the CO_2_ compensation point in the absence of daytime respiration (Yamori et al., 2010b; von Caemmerer and Evans, 2015). We used a typical value of 40 μmol mol^–1^ in our current study (Xiong et al., 2018). Respiration rate in the dark (*R*_*d*_) was considered to be half of the dark-adapted mitochondrial respiration rate as measured after 10 min of dark adaptation (Carriquí et al., 2015).

Based on the estimated *g*_*m*_, the chloroplast CO_2_ concentration (*C*_*c*_) was calculated according to the following equation (Long and Bernacchi, 2003; Warren and Dreyer, 2006):


(3) $$C_{\text{c}}=C_{\text{i}}-\frac{A_{\text{N}}}{{\text{g}}_{\text{m}}}$$

### Quantitative Limitation Analysis of *A*_*N*_

Relative photosynthetic limitations were assessed as follows (Grassi and Magnani, 2005):


(4) $$L_{\text{s}}=\frac{{\text{g}}_{\text{tot}}/{\text{g}}_{\text{s}}\times A_{\text{N}}/C_{\text{c}}}{{\text{g}}_{\text{tot}}+A_{\text{N}}/C_{\text{c}}}$$

(5) $$L_{\text{mc}}=\frac{{\text{g}}_{\text{tot}}/{\text{g}}_{\text{m}}\times A_{\text{N}}/C_{\text{c}}}{{\text{g}}_{\text{tot}}+A_{\text{N}}/C_{\text{c}}}$$

(6) $$L_{\text{b}}=\frac{{\text{g}}_{\text{tot}}}{{\text{g}}_{\text{tot}}+A_{\text{N}}/C_{\text{c}}}$$

where *L*_*s*_, *L*_*mc*_, and *L*_*b*_ represent the relative limitations of stomatal conductance, mesophyll conductance, and biochemical capacity, respectively, in setting the observed value of *A*_*N*_. *g*_*tot*_ is the total conductance of CO_2_ between the leaf surface and sites of RuBP carboxylation (calculated as 1/*g*_*tot*_ = 1/*g*_*s*_ + 1/*g*_*m*_).

### SPAD Index and Nitrogen Content Measurements

A handy chlorophyll meter (SPAD-502 Plus; Minolta, Tokyo, Japan) was used to nondestructively measure the SPAD index (relative content of chlorophyll per unit leaf area) of leaves used for photosynthetic measurements. Thereafter, leaf area was measured using a LI-3000A portable leaf area meter (Li-Cor, Lincoln, NE, United States). After leaf material was dried at 80°C for 48 h, dry weight was measured and leaf N content was determined with a Vario MICRO Cube Elemental Analyzer (Elementar Analysensysteme GmbH, Langenselbold, Germany) (Sakowska et al., 2018).

### Statistical Analysis

For each N treatment, five leaves form five independent plants were used for gas exchange and chlorophyll fluorescence measurements. One-way ANOVA and *t*-tests were used to determine whether significant differences existed between different treatments (α = 0.05). The software SigmaPlot 10.0 was used for graphing and fitting.

## Results

### Effect of Leaf N Content on Steady-State Physiological Characteristics Under High Light

The leaf N content in LN-, MN-, and HN-plants was 0.42 ± 0.03, 0.71 ± 0.3, and 1.2 ± 0.07 g m^–2^, respectively (Table 1). The HN-plants displayed the highest relative chlorophyll content, measured by SPAD value, followed by MN- and LN-plants. After 30 min light adaptation at 1,500 μmol photons m^–2^ s^–1^ and 400 μmol mol^–1^ CO_2_ concentration, HN-plants had the highest net CO_2_ assimilation rate (*A*_*N*_), stomatal conductance (*g*_*s*_), mesophyll conductance (*g*_*m*_), and ETR. Therefore, the steady-state photosynthetic capacities were significantly affected by leaf N content. Furthermore, HN-, MN-, and LN-plants showed slight difference in *g*_*s*_ but significant difference in *g*_*m*_, which indicates that *g*_*m*_ is more responsive to leaf N content than *g*_*s*_ in tomato.

**TABLE 1:** Physiological characteristics of leaves from plants grown under three different nutrient concentrations (low, medium and high nitrogen).

	Low N	Medium N	High N
Leaf N content (g m^–2^)	0.42 ± 0.03*a*	0.71 ± 0.3*b*	1.2 ± 0.07*c*
SPAD value	29.2 ± 1.2*a*	40.2 ± 1.7*b*	50.1 ± 1.7*c*
*A*_*N*_ (μmol m^–2^ s^–1^)	5.9 ± 0.3*a*	10.2 ± 0.29*b*	19.1 ± 0.67*c*
*g*_*s*_ (mol m^–2^ s^–1^)	0.22 ± 0.02*a*	0.25 ± 0.01*a*	0.31 ± 0.01*b*
*g*_*m*_ (mol m^–2^ s^–1^)	0.045 ± 0.002*a*	0.09 ± 0.007*b*	0.19 ± 0.01*c*
ETR (μmol m^–2^ s^–1^)	44 ± 2.7*c*	80 ± 2.0*b*	156 ± 3.9*a*

*All parameters were measured at 1,500 μmol photons m^–2^ s^–1^ and 400 μmol mol^–1^ CO_2_ concentration. Values are means ± SE (n = 5). Different letters indicate significant differences among different treatments.*

### Effects of Leaf N Content on Photosynthetic Induction Upon Transfer From Low to High Light

**FIGURE 1:**
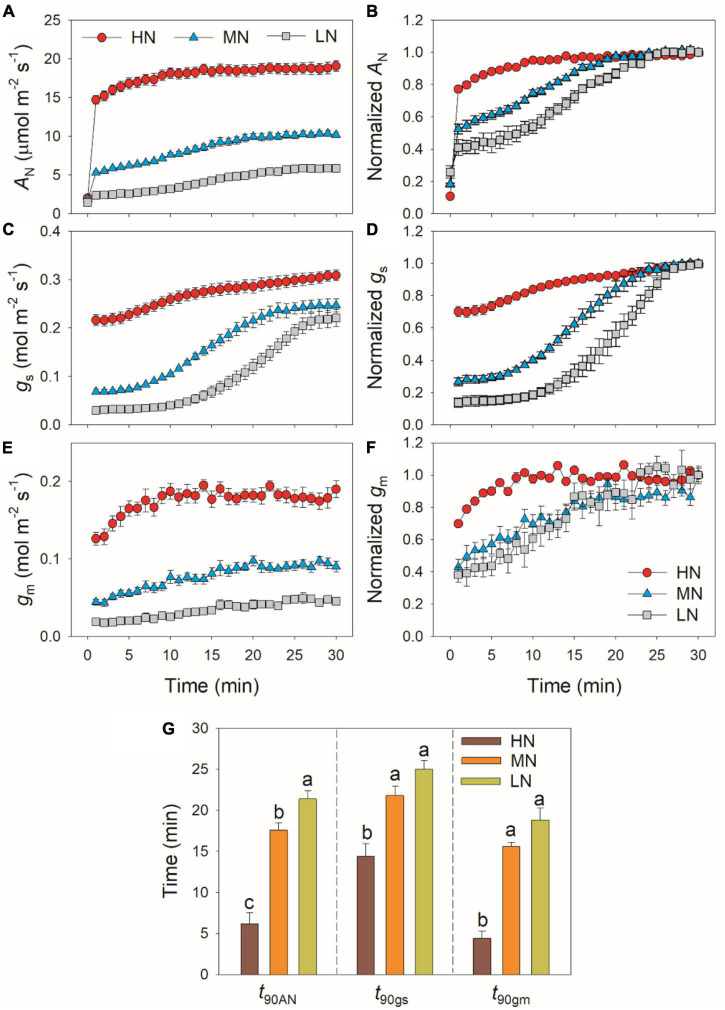
Induction response of net CO_2_ assimilation rate (*A*_*N*_) **(A,B)**, stomatal conductance (*g*_*s*_) **(C,D)** and mesophyll conductance (*g*_*m*_) **(E,F)**, and the time required to reach 90% of the maximum values of *A*_*N*_, *g*_*s*_ and *g*_*m*_ (*t*_90A*N*_, *t*_90g*s*_, *t*_90g*m*_) **(G)** after transition from 50 to 1,500 μmol photons m^–2^ s^–1^. *A*_*N*_, *g*_*s*_, and *g*_*m*_ were measured every 1 min. Values are means ± *SE* (*n* = 5). Different letters indicate significant differences among different treatments. The relative *A*_*N*_, *g*_*s*_, and *g*_*m*_ curves were obtained from the standardization against the maximum values after 30 min photosynthetic induction at high light. HN, MN, and LN represent tomato plants grown under high, medium, and low N concentrations, respectively.

**FIGURE 2:**
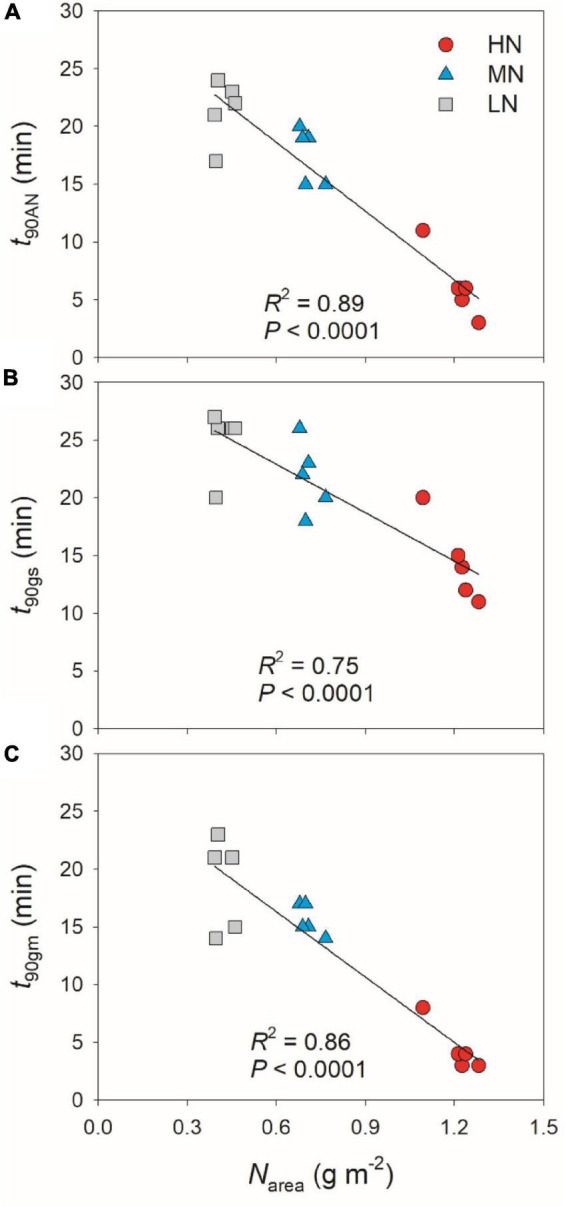
Effects of leaf N content on the time required to reach 90% of the maximum values of *A*_*N*_ **(A)**, *g*_*s*_ **(B)**, and *g*_*m*_ **(C)** (*t*_90A*N*_, *t*_90g*s*_, *t*_90g*m*_) after transition from 50 to 1,500 μmol photons m^–2^ s^–1^. HN, MN, and LN represent tomato plants grown under high, medium, and low N concentrations, respectively.

**FIGURE 3:**
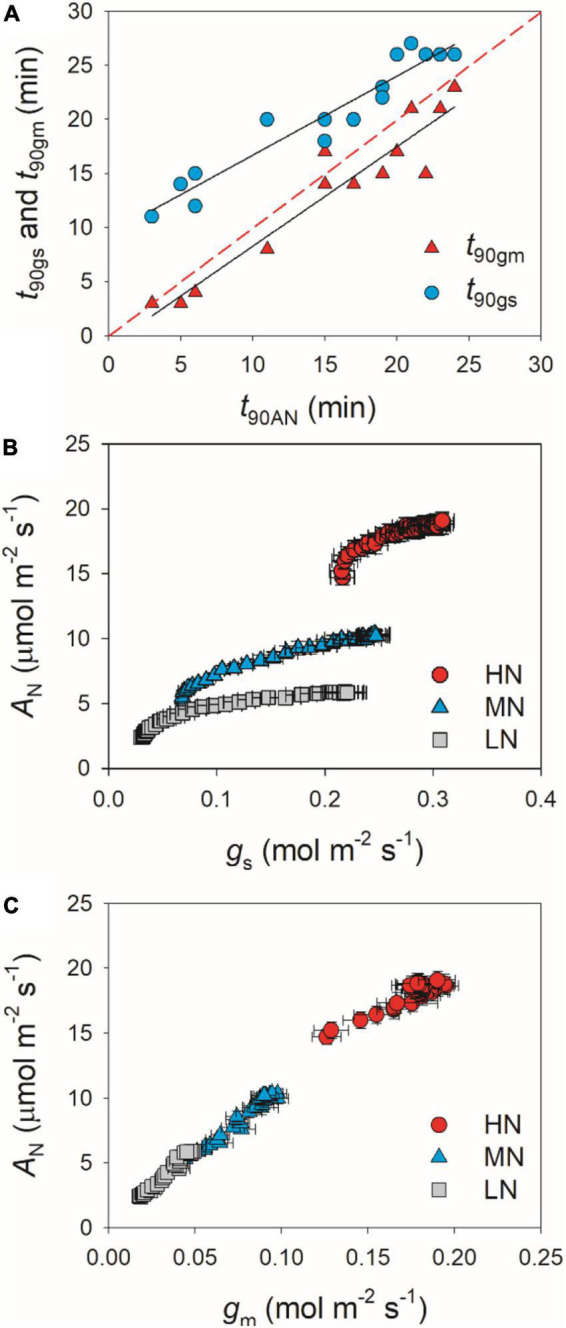
**(A)** Relationships between *t*_90A*N*_, *t*_90g*s*_, and *t*_90g*m*_ after transition from 50 to 1,500 μmol photons m^–2^ s^–1^. **(B,C)** Relationships between *g*_*s*_, *g*_*m*_, and *A*_*N*_ after transition from 50 to 1,500 μmol photons m^–2^ s^–1^. Values are means ± SE (*n* = 5). HN, MN, and LN represent tomato plants grown under high, medium, and low N concentrations, respectively.

During this photosynthetic induction after 5 min of shadefleck, HN-plants showed the highest induction speeds of *A*_*N*_, *g*_*s*_, and *g*_*m*_, followed by MN- and LN-plants (Figure 1). The time required to reach 90% of the maximum *A*_*N*_ (*t*_90A*N*_) significantly increased with the decrease in leaf N content (Figure 1G). The time required to reach 90% of the maximum *g*_*s*_ and *g*_*m*_ (*t*_90g*s*_ and *t*_90g*m*_, respectively) was significantly shorter in HN-plants than MN- and LN-plants, whereas *t*_90g*s*_ and *t*_90g*m*_ did not differ significantly between MN- and LN-plants (Figure 1G). Interestingly, *t*_90g*m*_ was lower than *t*_90g*s*_ in all plants. The higher *t*_90g*s*_ and *t*_90A*N*_ in MN- and LN-plants were

partially related to the relatively lower initial *g*_*s*_ prior to light change (Supplementary Figure 1). Within the first 15 min after transition from low to high light, all plants showed similar intrinsic water use efficiency (iWUE) (Supplementary Figure 2). However, during prolonged photosynthetic induction, HN-plants displayed much higher iWUE than MN- and LN-plants (Supplementary Figure 2). Further analysis found that leaf N content was negatively correlated with *t*_90A*N*_, *t*_90g*s*_, and *t*_90g*m*_ (Figure 2). Therefore, leaf N content plays a crucial role in affecting the induction responses of *A*_*N*_, *g*_*s*_, and *g*_*m*_ after transition from low to high light. The comparative extent of the reductions of *t*_90A*N*_ was more correlated to *t*_90g*m*_ than *t*_90g*s*_ (Figure 3A). Furthermore, the change in *A*_*N*_ during photosynthetic induction was more related to *g*_*m*_ than *g*_*s*_ (Figures 3B,C). These results suggest that, upon transfer from low to high light, *g*_*m*_ plays a more important role in determining the induction response of *A*_*N*_ than *g*_*s*_.

### Effects of Leaf N Content on Intercellular and Chloroplast CO_2_ Concentrations Upon Transfer From Low to High Light

**FIGURE 4:**
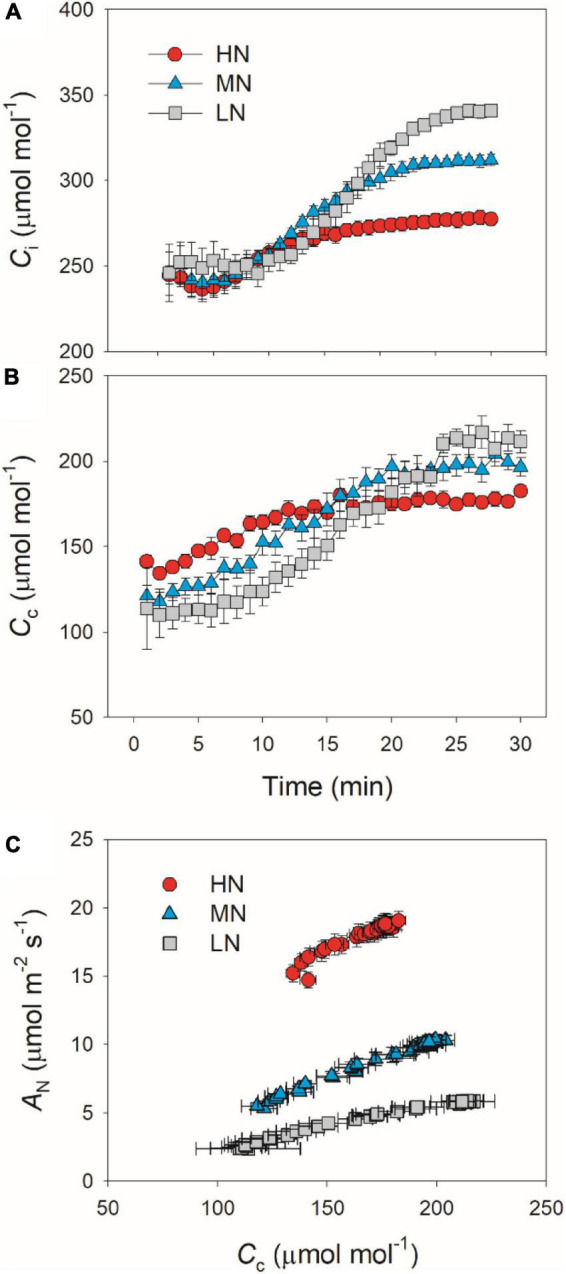
**(A,B)** Response of intercellular CO_2_ concentration (*C*_*i*_) and chloroplast CO_2_ concentration (*C*_*c*_) after transition from 50 to 1,500 μmol photons m^–2^ s^–1^. **(C)** Relationship between *C*_*c*_ and *A*_*N*_ after transition from 50 to 1,500 μmol photons m^–2^ s^–1^. Values are means ± SE (*n* = 5). HN, MN, and LN represent tomato plants grown under high, medium, and low N concentrations, respectively.

**FIGURE 5:**
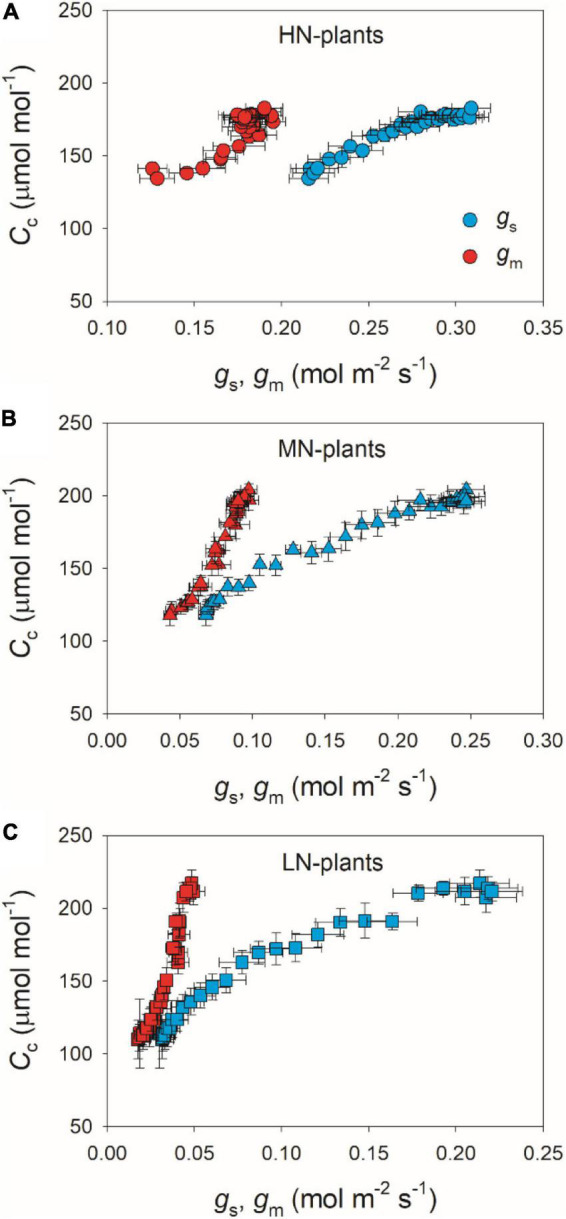
Relationships between *g*_*s*_, *g*_*m*_ and *C*_*c*_ after transition from 50 to 1,500 μmol photons m^–2^ s^–1^ in HN-plants **(A)**, MN-plants **(B)**, and LN-plants **(C)**. Values are means ± SE (*n* = 5). HN, MN, and LN represent tomato plants grown under high, medium, and low N concentrations, respectively.

We calculated the response kinetics of intercellular (*C*_*i*_) and chloroplast CO_2_ concentration (*C*_*c*_) using *A*_*N*_, *g*_*s*_, and *g*_*m*_. After transitioning from low to high light, *C*_*i*_ and *C*_*c*_ gradually increased in all plants (Figure 4). HN-plants had the lowest values of *C*_*i*_ and *C*_*c*_ after photosynthetic sufficient photosynthetic induction. The change in *A*_*N*_ during photosynthetic induction was tightly and positively correlated with *C*_*c*_ in all plants, which suggests the importance of *C*_*c*_ in determining *A*_*N*_. Because *C*_*c*_ can be affected by *g*_*s*_ and *g*_*m*_, we analyzed the relationships between *C*_*c*_, *g*_*s*_, and *g*_*m*_. Compared with *g*_*s*_, a smaller change in *g*_*m*_ could result in a larger change in *C*_*c*_ (Figure 5), which suggests that the change of *C*_*c*_ upon transfer from low to high light was more determined by *g*_*m*_ than *g*_*s*_.

### Effects of Leaf N Content on Relative Limitations of Photosynthesis Upon Transfer From Low to High Light

**FIGURE 6:**
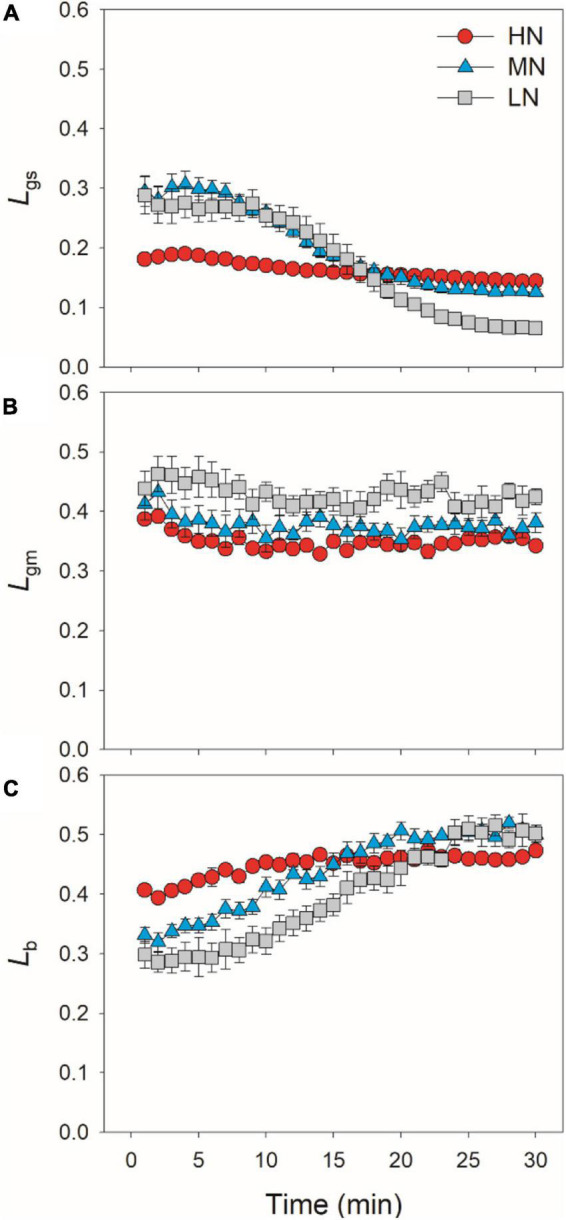
Quantitative analysis of the relative limitations of *g*_*s*_ **(A)**, *g*_*m*_ **(B)** and biochemical factors **(C)** imposed to photosynthesis after transition from 50 to 1,500 μmol photons m^–2^ s^–1^. Values are means ± SE (*n* = 5). HN, MN, and LN represent tomato plants grown under high, medium, and low N concentrations, respectively.

**FIGURE 7:**
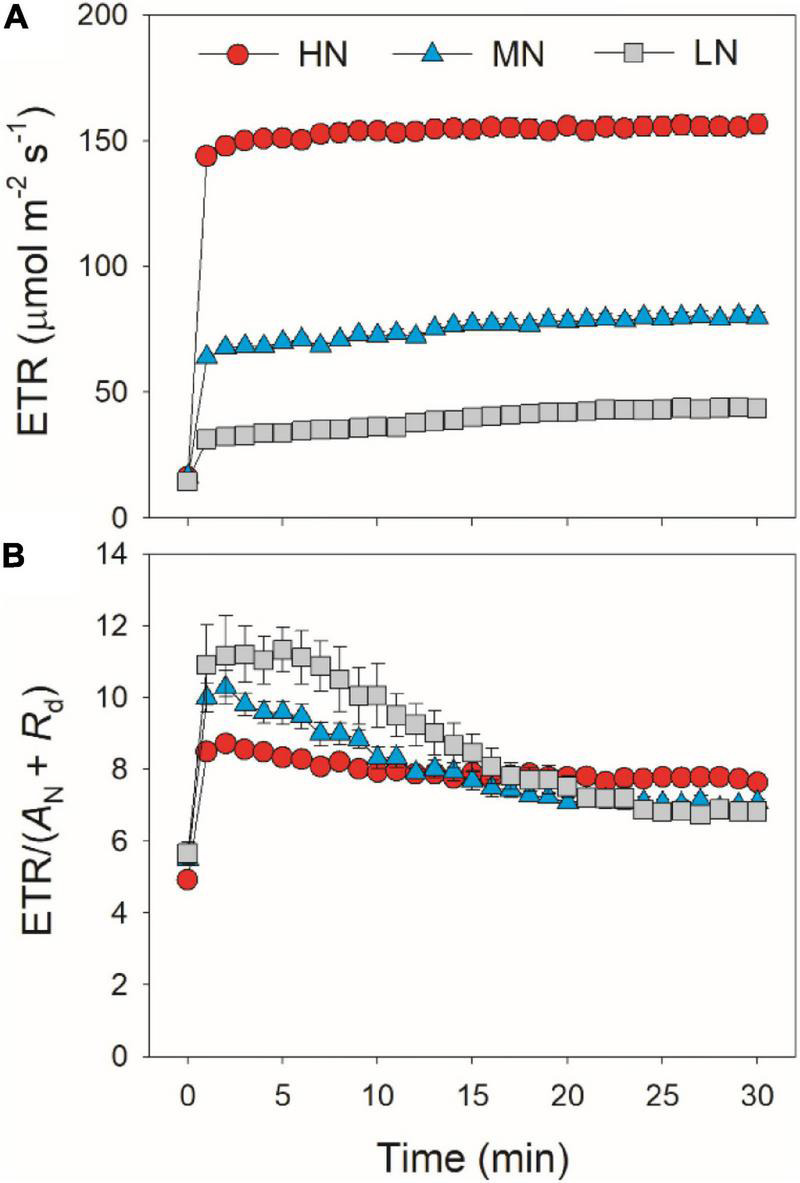
Response of ETR **(A)** and the ratio of ETR to (*A*_*N*_ + *R*_*d*_) **(B)** after transition from 50 to 1,500 μmol photons m^–2^ s^–1^. Values are means ± SE (*n* = 5). HN, MN, and LN represent tomato plants grown under high, medium, and low N concentrations, respectively.

After transition from low to high light, the limitations of photosynthesis by *g*_*s*_ (*L*_*gs*_), *g*_*m*_ (*L*_*gm*_), and biochemical factors (*L*_*b*_) changed slightly in HN-plants (Figure 6). In MN- and LN-plants, *L*_*gs*_ gradually decreased over time. Within the first 15 min, *L*_*gs*_ was lower in HN-plants than MN- and LN-plants. However, the LN-plants had the lowest *L*_*gs*_ after sufficient photosynthetic induction. *L*_*gm*_ was also maintained stable during whole photosynthetic induction in MN- and LN-plants, but *L*_*b*_ gradually increased from 0.3 to 0.5 in them. Therefore, leaf N content could affect the kinetics of relative limitations of photosynthesis during photosynthetic induction after transfer from low to high light. To explore whether the induction of *A*_*N*_ is limited by photosynthetic electron transport, we estimated the dynamic change in ETR. Upon a sudden increase in illumination, ETR rapidly increased and the ETR/(*A*_*N*_ + *R*_*d*_) ratio first increased and then gradually decreased in all plants (Figure 7). These results indicated that the activation speed of ETR was much faster than that of *A*_*N*_. Therefore, during photosynthetic induction, the limitation of ETR imposed to *A*_*N*_ was negligible in all samples.

## Discussion

Leaf N content plays an important role in determining photosynthesis, plant growth, and crop productivity (Makino, 2011). Under natural field conditions, FL and N deficiency usually occurs concomitantly. However, it is unknown how FL and N deficiency interacts to influence photosynthetic physiology in crop plants. In this study, we here for the first time examined the effects of leaf N content on photosynthetic induction after transition from low to high light in tomato. We found that leaf N content significantly affected the induction responses of *g*_*s*_ and *g*_*m*_ and thus affected induction kinetics of *A*_*N*_. However, the activation speed of photosynthetic electron flow was not influenced by leaf N content. Therefore, the effect of leaf N content on photosynthetic induction was more attributed to the induction kinetics of diffusional conductance rather than the activation speed of electron transport.

In addition to steady-state photosynthetic capacity under high light, the photosynthetic responses to the changes in illumination significantly affect the carbon gain and plant biomass (Adachi et al., 2019; Kimura et al., 2020; Zhang et al., 2020). Many previous studies have documented that leaf N content influences the steady-state photosynthetic performances under high light (Evans and Terashima, 1988; Makino and Osmond, 1991), but few is known about the influence of leaf N content on photosynthetic induction under FL conditions. Similar to previous studies, the maximum steady-state *A*_*N*_ under high light significantly declined with the decrease in leaf N content (Table 1). Moreover, we here found that, after transition from low to high light, the HN-plants showed much faster induction response of *A*_*N*_ than MN- and LN-plants (Figure 1). The time required to reach 90% of the steady state of photosynthesis (*t*_90A*N*_) was negatively correlated to leaf N content (Figure 2). Therefore, leaf N content significantly affects the photosynthetic induction after transition from low to high light in tomato. This finding is similar to the photosynthetic induction of dark-adapted leaves among canola genotypes (*Brassica napus* L.) (Liu et al., 2021), but was inconsistent with the phenomenon in soybean (Li et al., 2020) and *Panax notoginseng* (Chen et al., 2014). In soybean, the induction rate of *A*_*N*_ under high light after shading for 5 min was very fast (Pearcy et al., 1996; Li et al., 2020). Furthermore, this fast photosynthetic induction in soybean was not affected by leaf N content (Li et al., 2020). In the shade-establishing plant *Panax notoginseng*, the higher leaf N content in shade leaves was accompanied with slower photosynthetic induction rate than sun leaves (Chen et al., 2014). Therefore, the effect of leaf N content on fast photosynthetic induction following shade fleck depends on the species and on growth conditions. In MN- and LN-plants of tomato, the delayed induction of *A*_*N*_ caused a larger loss of carbon gain under FL. This finding provides insight into why plants grown under low N concentrations display reduction in plant biomass under natural field FL conditions.

After transition from low to high light, the time to reach the maximum *C*_*c*_ was less in HN-plants than MN- and LN-plants (Figure 4). Furthermore, tight and positive relationships were found between *C*_*c*_ and *A*_*N*_ in all plants (Figure 4). These results suggested that the induction response of *A*_*N*_ was largely determined by the change of CO_2_ concentration in the site of RuBP carboxylation. The value of *C*_*c*_ in a given leaf is largely affected by CO_2_ diffusional conductance, which includes *g*_*s*_ and *g*_*m*_ (Sagardoy et al., 2010; Carriquí et al., 2015; Yang Z.-H. et al., 2018). However, it is unclear whether the photosynthetic induction of *A*_*N*_ upon transfer from low to high light is more determined by the induction response of *g*_*s*_ or *g*_*m*_. We found that the induction responses of *g*_*s*_ and *g*_*m*_ were largely delayed in MN- and LN-plants than HN-plants (Figure 1), and the induction rates of *g*_*s*_ and *g*_*m*_ were negatively correlated with leaf N content (Figure 2). Furthermore, the change of *C*_*c*_ during photosynthetic induction was more related to *g*_*m*_ rather than *g*_*s*_ (Figure 5), which pointing out the important role of *g*_*m*_ response in determining *C*_*c*_ upon transfer from low to high light. Therefore, the delayed photosynthetic induction of *A*_*N*_ in plants grown under low N concentrations was more attributed to the slower induction response of *g*_*m*_ than *g*_*s*_.

In HN-plants of tomato, photosynthetic limitations by *g*_*s*_, *g*_*m*_, and biochemical factors changed slightly upon transfer from low to high light. Meanwhile, *g*_*s*_ imposed to the smallest limitation to *A*_*N*_, owing to the high levels of *g*_*s*_ (Figure 6). Therefore, improving the induction response of *g*_*s*_ might have a minor factor for improving photosynthesis under FL in HN-plants of tomato under optimal conditions (Kaiser et al., 2020). By comparison, increased *g*_*s*_ has a significant effect on photosynthetic CO_2_ assimilation under FL in *Arabidopsis thaliana* and rice (Kimura et al., 2020; Yamori et al., 2020). These results indicate that the effects of altered *g*_*s*_ kinetics on photosynthesis under FL are species-dependent. In MN- and LN-plants, the relatively slower kinetics of *g*_*s*_ led to a higher *L*_*gs*_ of *A*_*N*_ during the initial 15 min after transition from low to high light (Figure 6). Therefore, altered *g*_*s*_ kinetics would have more significant effects on photosynthetic carbon gain in crop plants grown under low N concentrations.

Many previous studies have indicated that *g*_*m*_ act as a major limitation for steady-state *A*_*N*_ under high light in many angiosperms (Peguero-Pina et al., 2017; Théroux-Rancourt and Gilbert, 2017; Yang Y.-J. et al., 2018; Huang et al., 2019). Increasing *g*_*m*_ has been thought to be a potential target for improving crop productivity and water use efficiency under constant high light (Flexas et al., 2013; Gago et al., 2016). However, the limitation of *g*_*m*_ imposed to *A*_*N*_ under FL is poorly understood. Upon transition from dark to light, the induction response of *g*_*m*_ was much faster than that of *g*_*s*_, which leads to the smallest limitation of *g*_*m*_ imposed to *A*_*N*_ in *Arabidopsis thaliana* and tobacco (Sakoda et al., 2021). Consequently, one concluded that altering *g*_*m*_ kinetics would have less impact on *A*_*N*_ under FL. However, we found that, after transfer from low to high light, *L*_*gm*_ was higher than *L*_*gs*_ in tomato plants (Figure 6). Furthermore, the time to reach 90% of *A*_*N*_ was closer to that of *g*_*m*_ rather than that of *g*_*s*_ (Figure 3). Therefore, altering *g*_*m*_ kinetics would significantly influence *A*_*N*_ upon transfer from low to high light, at least in tomato. These results suggested that the photosynthetic limitation upon transfer from low to high light was largely different from the photosynthetic induction during illumination of dark-adapted leaves. Improving the induction rate of *g*_*m*_ has a potential to enhance carbon gain and plant biomass under natural FL conditions.

A recent study reported that, if RuBP regeneration limitation was assumed, electron transport imposed the greatest limitation to *A*_*N*_ during illumination of dark-adapted leaves (Sakoda et al., 2021). Based on this result, it is hypothesized that increased activation of electron transport has the potential to enhance carbon gain under naturally FL environments. Controversially, this study indicated that electron transport was rapidly activated upon transfer from low to high light. After transition from low to high light, the ETR/(*A*_*N*_ + *R*_*d*_) value rapidly increased to the peak within 1–2 min and then gradually decreased over time (Figure 7). These results indicated that, upon transfer from low to high light, the induction response of electron transport was much faster than that of *A*_*N*_, which was consistent with the photosynthetic performance in rice (Yamori et al., 2016b). Therefore, induction response of *A*_*N*_ after transition from low to high light was hardly limited by electron transport in tomato. The effect of electron transport on *A*_*N*_ upon transition from low to high light is largely different from that upon transition from dark to light. Therefore, to improve photosynthesis under FL in tomato, more attention should be focused on the induction kinetics of CO_2_ diffusional conductance rather than the activation of electron transport.

## Conclusion

We studied the effects of leaf N content on photosynthetic induction after transfer from low to high light in tomato. The induction speeds of *A*_*N*_, *g*_*s*_, and *g*_*m*_ significantly decreased with the decrease in leaf N content. Such delayed photosynthetic induction in plants grown under low N concentration caused a larger loss of carbon gain under FL conditions, which further explained why N deficiency reduced plant biomass under natural FL environments. After transition from low to high light, increasing the induction responses of *g*_*s*_ and *g*_*m*_ has the potential to improve *A*_*N*_ in tomato, especially when plants are grown under low N concentration, whereas photosynthetic induction of *A*_*N*_ was hardly limited by electron transport. Therefore, altering induction kinetics of CO_2_ diffusional conductance is likely the most effective target for improving photosynthesis under FL conditions in tomato.

## Data Availability Statement

The raw data supporting the conclusions of this article will be made available by the authors, without undue reservation.

## Author Contributions

WH and S-BZ designed the study. HS performed the photosynthetic measurements. HS, Y-QZ, and WH performed the data analysis. WH wrote the first draft of the manuscript, which was extensively edited by all authors.

## Conflict of Interest

The authors declare that the research was conducted in the absence of any commercial or financial relationships that could be construed as a potential conflict of interest.

## Publisher’s Note

All claims expressed in this article are solely those of the authors and do not necessarily represent those of their affiliated organizations, or those of the publisher, the editors and the reviewers. Any product that may be evaluated in this article, or claim that may be made by its manufacturer, is not guaranteed or endorsed by the publisher.

## Supplementary Material

Supplementary Figure 1 Relationships between *t*_90AN_ **(A)**, *t*_90gs_ **(B)**, and the initial g_s_ prior to light change. HN, MN, and LN represent tomato plants grown under high, medium, and low N concentrations, respectively.

Supplementary Figure 2 Response of intrinsic water use efficiency (iWUE) after transition from 50 to 1,500 μmol photons m^−2^ s^−1^. Values are means ± SE (*n* = 5). HN, MN, and LN represent tomato plants grown under high, medium, and low N concentrations, respectively.
