# Supplementary figures and images for: Photosynthetic Induction Under Fluctuating Light Is Affected by Leaf Nitrogen Content in Tomato
Source: Front Plant Sci. 2022 Feb 17;13:835571. doi: 10.3389/fpls.2022.835571 (PMC8891375; doi:10.3389/fpls.2022.835571)

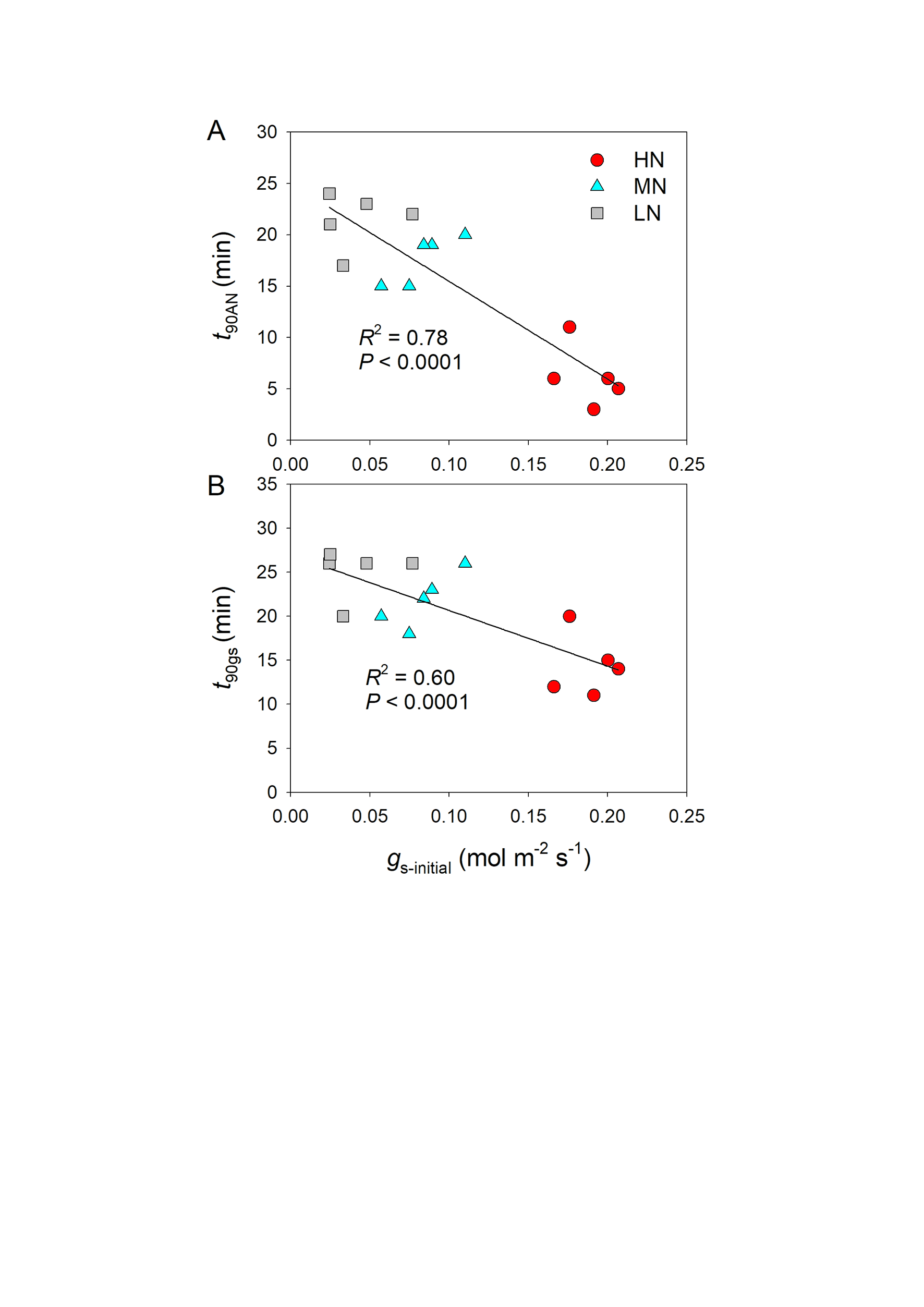

Supplement: Supplementary Figure 1 — Relationships between t90AN (A), t90gs (B), and the initial gs prior to light change. HN, MN, and LN represent tomato plants grown under high, medium, and low N concentrations, respectively. [file Image_1.tif]

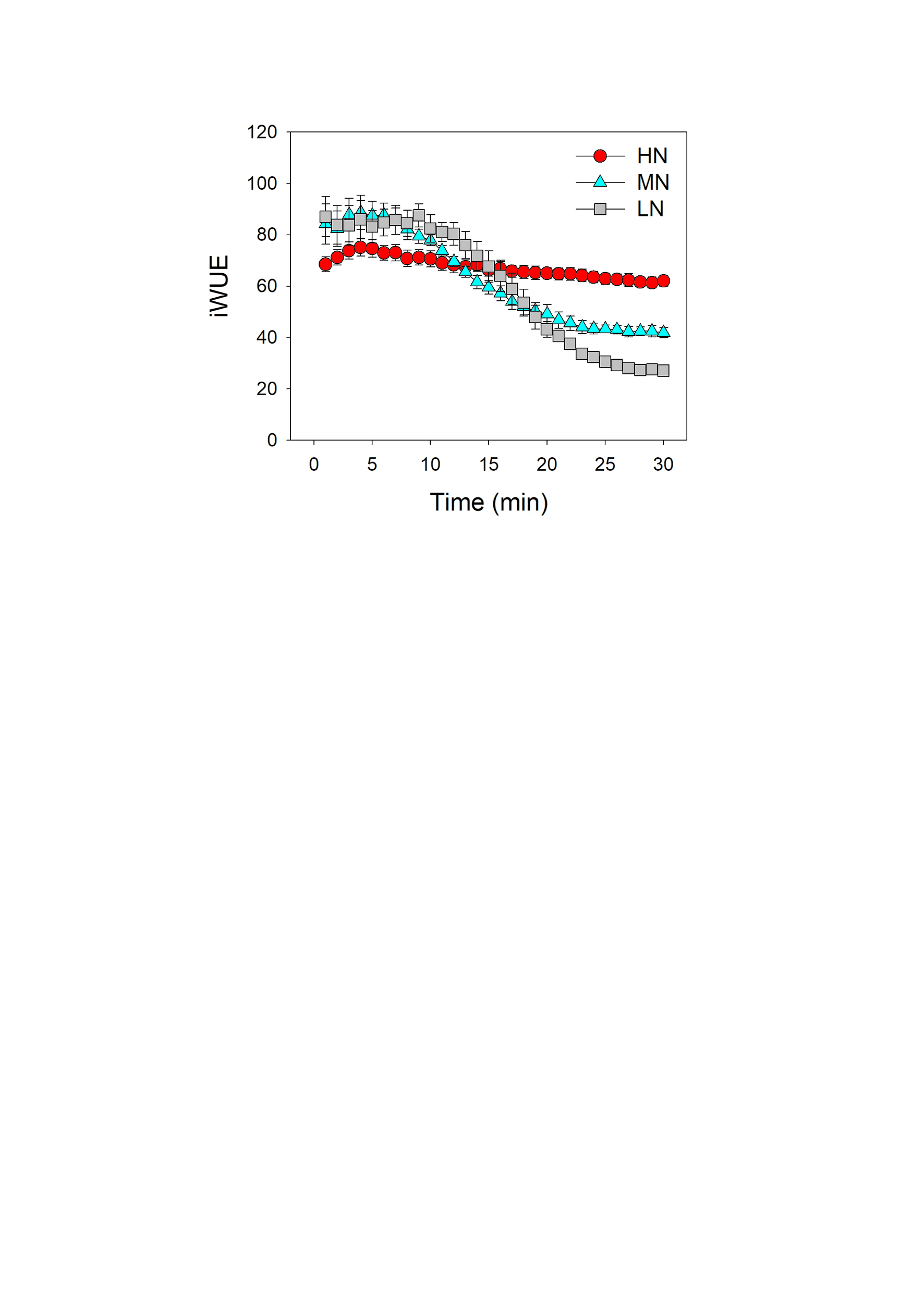

Supplement: Supplementary Figure 2 — Response of intrinsic water use efficiency (iWUE) after transition from 50 to 1,500 μmol photons m−2 s−1. Values are means ± SE (n = 5). HN, MN, and LN represent tomato plants grown under high, medium, and low N concentrations, respectively. [file Image_2.tif]
